# Cristae Architecture as a Metabolic Checkpoint in Effector T Cells — Implications for Cancer Immunity

**DOI:** 10.33696/cancerimmunol.8.120

**Published:** 2026

**Authors:** Prakash Jeysankar, Sonal Srikanth, Beibei Wu, Yousang Gwack

**Affiliations:** 1 Department of Physiology, David Geffen School of Medicine, UCLA, Los Angeles, CA 90095, USA

**Keywords:** Effector T cells, Mitochondria, Cristae, Reactive oxygen species

## Abstract

Effector T cells rely on tightly coordinated metabolic and epigenetic programs to sustain immune function. Emerging evidence highlights a central role for mitochondria in integrating these programs through nutrient utilization and regulation of metabolite flux. The electron transport chain (ETC), localized to the inner mitochondrial membrane, directs cellular metabolism toward oxidative phosphorylation. The efficiency of ETC activity is governed by the highly folded architecture of the inner mitochondrial membrane into cristae. Although mitochondrial metabolism is well recognized as a key determinant of cellular metabolic states, the regulatory roles of cristae-organizing structural proteins, particularly in T cells, remain poorly defined. Our recent study identifies the inner mitochondrial membrane protein TMEM11 as a critical structural determinant of cristae organization and demonstrates how cristae integrity governs effector T cell function by controlling oxidative phosphorylation and metabolite flux. TMEM11 deficiency disrupts cristae architecture in T cells without affecting mitochondrial biogenesis or cell viability. Mechanistically, loss of TMEM11 impairs ETC function, leading to elevated mitochondrial reactive oxygen species (mtROS), which diverts acetyl-CoA away from histone acetylation toward fatty acid synthesis, thereby suppressing cytokine production. Collectively, these findings reveal a structural-metabolic-epigenetic axis that is essential for effector T cell immunity and suggest potential relevance for T cell-mediated cancer therapy.

## Introduction

The mitochondrial contribution to T cell immunity is traditionally viewed through metabolic pathways such as oxidative phosphorylation, glycolysis, and substrate utilization. Yet mitochondria are not static metabolic devices; they are structurally dynamic organelles whose architecture profoundly influences their function [1]. Nowhere is this more relevant than in the tumor microenvironment, where persistent hypoxia, nutrient competition, and oxidative pressure disrupt T cell metabolism, contributing to exhaustion and impaired anti-tumor responses [2,3].

Our recent work brings mitochondrial inner membrane architecture into the immunological spotlight. We found that TMEM11, a component linked to the mitochondrial inner membrane cristae organizing system (MICOS) complex, is essential for maintaining cristae architecture in effector T cells, particuarly Th1 cells [4]. Loss of TMEM11 leads to profound changes in mitochondrial structure, respiratory activity, and redox balance, which in turn reshape the epigenetic landscape of effector T cells. These results introduce cristae morphology as a previously unrecognized checkpoint governing immune cell function. In this commentary, we discuss what is known about the structural components of the mitochondrial cristae in T cells and speculate on how these studies can provide mechanistic insights into the mitochondrial defects widely observed in tumor-infiltrating lymphocytes (TILs).

### TMEM11 is a structural component of mitochondrial cristae

**Figure 1.**
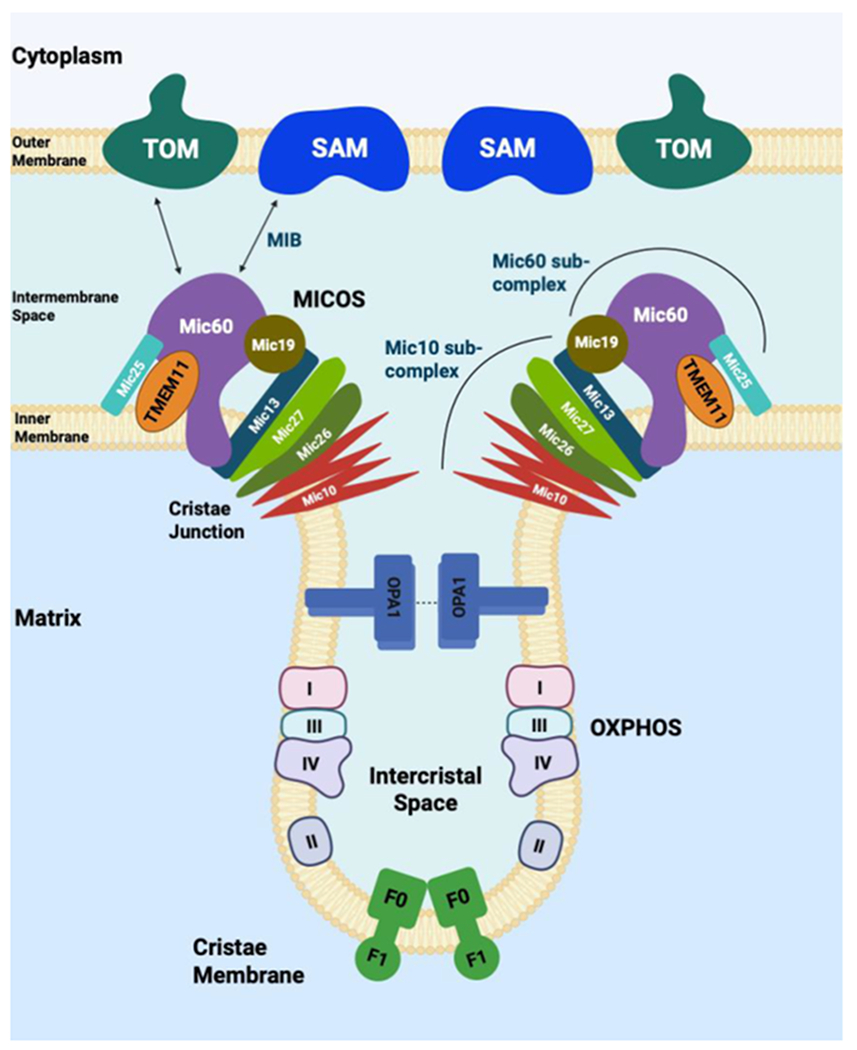
Structural components of mitochondrial cristae. The mitochondrial contact site and cristae organizing system (MICOS) complex is known to stabilize cristae junctions by providing membrane curvature as well as establishing contact sites with other membrane protein complexes. For the ease of readability, the MICOS subunits are indicated with their universal nomenclature rather than the mammalian homologs such as MIC10/MINOS1, MIC60/MITOFILIN, MIC19/CHCHD3, MIC25/CHCHD6, MIC13/QIL1, MIC26/APOO and MIC27/APOOL. MIB is the mitochondrial intermembrane space bridging complex resulting from the interaction between the SAM (sorting and assembly machinery) and MICOS complexes. Translocase of the outer mitochondrial membrane (TOM) and voltage dependent anion channel (VDAC) are also known to interact with the MICOS complexes. TMEM11 was shown to physically interact with MIC60. Physical interaction between protein complexes is indicated by solid arrows. Homotypic interactions between optic atrophy 1 (OPA1) mediate fusion of the inner membrane to determine cristae width and is also involved in mitochondria fusion. The mitochondrial F_1_-F_0_ ATP synthase (complex V) generates a strong positive curvature at the cristae rims by forming V-shaped dimers that can bend membrane by forming oligomers. The electron transport chain (ETC) CI, CII, CIII and CIV complexes reside in the lateral surfaces of the cristae.

Mitochondria are enclosed by an outer and an inner membrane. The outer mitochondrial membrane (OMM) separates mitochondria from the cytosol and contains the voltage-dependent anion channel (VDAC), the sorting and assembly machinery (SAM) complex, proteins involved in mitochondrial dynamics (e.g., mitofusin 1, mitofusin 2, and fission protein 1), and components of the apoptotic pathway, including BAK. The inner mitochondrial membrane (IMM) runs parallel to the OMM at regions known as inner boundary membranes and extends into the mitochondrial matrix to form cristae structures [5]. Mitochondrial cristae are specialized compartments for limiting the diffusion of molecules that are important for the electron transport chain (ETC) and enable efficient electron flow. The MICOS complex, OPA1, and F_1_F_0_-ATP synthase (complex V) are essential for cristae formation [6,7]. The MICOS complex, composed of Mitofilin/Fcj1 (MIC60) and MINOS1/Mio10 (MIC10) subcomplexes, is essential for the formation of cristae junctions [6,7]. In yeast, these subcomplexes form two distinct and independent MICOS-organizing assemblies that are linked by MIC19 [8]. MIC60 forms the mitochondrial intermembrane bridging complex that connects with the SAM50 protein in the OMM through MIC19, while MIC10 controls cristae shape to establish inner membrane curvature (Figure 1). The mitochondrial fusion protein, OPA1, multimerizes with itself and maintains the width of cristae junctions. F_1_F_0_-ATP synthase localizes to the tip of the cristae to form membrane curvature. The other ETC complexes reside in the lateral surfaces of the cristae.

TMEM11 was originally identified in *Drosophila* as a protein critical for mitochondrial cristae morphogenesis, and flies deficient in PMI (the *Drosophila* ortholog of TMEM11) exhibit disrupted cristae architecture, reduced respiration capacity, and a shortened lifespan [9,10]. However, the localization of TMEM11 on the mitochondrial membrane has been controversial. CFP-fused PMI showed localization to IMM cristae by confocal and electron microscopy [9]. Using protease protection assays on purified mitochondria from HeLa cells in the same study, TMEM11 was resistant to proteolytic degradation, in contrast to VDAC, which was readily degraded due to its OMM localization, supporting an inner mitochondrial membrane localization of TMEM11 [9]. Further, proteomic analysis of several MICOS components identified TMEM11 as an interactor of several MICOS complex proteins including MIC19, MIC27, and MIC60, of which endogenous interaction was verified with MIC60 in HEK293T cells, further supporting its role in cristae formation [11]. However, an early study using high throughput yeast two-hybrid interactome data implicated TMEM11 as interactor of OMM BINP3/BNIP3L proteins [12]. Another study reported that a majority of TMEM11 localized to the outer mitochondrial membrane in U2OS cells, where it formed complexes with BNIP3 and BNIP3L to regulate mitophagy, as demonstrated by two-dimensional blue native PAGE, immunoprecipitation, and electron microscopy of GFP-tagged TMEM11 [13]. Notably, a smaller fraction of TMEM11 was also found to associate with the MICOS complex at the inner mitochondrial membrane. Interestingly, *TMEM11* deletion in U2OS cells did not affect mitochondrial respiratory capacity in this study, in contrast to findings from previous reports. By comparison, in our recent work identifying TMEM11 as a genetic determinant of Th1 effector function, we validated a physical interaction between TMEM11 and endogenous MIC60, supporting a role for TMEM11 in the regulation of mitochondrial cristae architecture [4]. *Tmem11*^−/−^ Th1 cells exhibited enlarged mitochondria with profound defects in cristae architecture and respiratory capacity, indicating a predominant role for TMEM11 in regulating IMM structure in Th1 cells. Notably, loss of TMEM11 in T cells did not affect mitochondrial abundance, suggesting a minimal contribution to mitophagy. Collectively, despite the use of broadly comparable biochemical and imaging approaches, the divergent findings across studies suggest that TMEM11 may adopt distinct sub-mitochondrial localizations, potentially partitioning between the inner and outer mitochondrial membranes to form functionally distinct protein complexes. Such context-dependent localization could differentially influence mitochondrial respiration or mitophagy across cell types and metabolic states. Definitive resolution of TMEM11 localization and function will require future studies combining advanced immunoelectron microscopy using highly specific antibodies against endogenous TMEM11 with parallel assessments of mitochondrial respiratory capacity across diverse cellular contexts.

### Cristae structure controls respiratory function and mitochondrial reactive oxygen species in T cells

Mitochondrial cristae morphology is intricately linked to metabolism in T cells. Naïve T cells are metabolically quiescent and predominantly rely on oxidative phosphorylation for development and survival [14]. Effector T cells on the other hand rapidly increase glucose uptake and glycolysis to generate metabolic intermediates necessary for cell proliferation. The mitochondria from effector T cells have been shown to exhibit a fragmented morphology, with loosely packed cristae [15,16]. Memory T cells on the other hand become less active metabolically and exhibit elongated mitochondria with tightly packed cristae. Thus mitochondria shape and cristae organization vary with the metabolic status of T cells.

Consistent with the previous view, TMEM11-deficient effector Th1 cells show enlarged mitochondria with elongated and widened cristae but reduced cristae number, despite normal expression of ETC components [4,9,10]. This altered architecture results in diminished oxygen consumption, reduced mitochondrial membrane potential, and a marked increase in mitochondrial ROS (mtROS), particularly during TCR stimulation [4]. The elevated mtROS level is consistent with increased electron leak, likely resulting from disorganized cristae that disrupt the spatial organization of respiratory supercomplexes. Notably, although all major effector T cell subsets — including Th1, Th2, regulatory T cells, and Th17 cells — express comparable levels of TMEM11 protein, deletion of *Tmem11* selectively impairs the function of Th1 cells. While TMEM11 deficiency results in similar reductions in mitochondrial metabolic parameters, including respiration, across these subsets, mtROS accumulation is disproportionately elevated only in TMEM11-deficient Th1 cells [4]. This increase in mtROS directly compromises Th1 effector function, as evidenced by impaired IFNγ production. This susceptibility highlights a fundamental constraint in the Th1 antioxidant program, rendering these cells uniquely fragile in the face of mitochondrial structural dysfunction — a paradigm that warrants further mechanistic dissection.

### Influence of mtROS on T cell epigenetics

A central insight from our study is that excessive mtROS alters usage of acetyl-CoA, an essential metabolite that links mitochondrial metabolism to epigenetic regulation in Th1 cells. Increased pyruvate influx via glycolysis to mitochondria enhances the generation of citrate, which is exported to the cytoplasm and converted to acetyl-CoA, a cofactor for histone acetylation [17]. Deleting either the citrate transporter, Slc25a1, or ATP-citrate lyase (ACLY) in activated CD4^+^ T cells decreased H3K9 acetylation and *Ifng* transcription, emphasizing the role of mitochondria in this pathway [18]. TMEM11 deficiency disrupts this balance not by reducing acetyl-CoA production, but by redirecting acetyl-CoA away from histone acetylation toward lipid synthesis [4]. TMEM11-deficient Th1 cells display reduced H3K9 acetylation at promoters of *Ifng, Ccl3, Ccl4, Ccl5*, and *Ccl9*, accompanied by dramatic accumulation of neutral lipids [4]. Both antioxidant treatment and acetate supplementation restore histone acetylation and cytokine expression, confirming that mtROS drives the metabolic rerouting to lipid synthesis, away from histone acetylation. Pharmacologic inhibition of acetyl-CoA carboxylase or fatty acid synthase also rescues effector function by increasing acetyl-CoA availability for chromatin modification. Together, these findings define a mtROS-dependent epigenetic checkpoint: when cristae disruption elevates mtROS beyond a threshold, acetyl-CoA is diverted toward fatty acid synthesis, and the epigenetic activation of effector loci is suppressed. This mechanism represents a metabolic brake designed perhaps to protect cells under oxidative stress, but at the cost of attenuating effector immunity.

Currently, very little is known about the role of mitochondrial cristae architecture in effector T cell function. One recent study examined the role of mitochondrial cristae architecture among naïve and effector T cells and found that Th17 cells uniquely harbor fused mitochondria with tightly packed cristae [19]. Using mice with T cell-specific deletion of *Opa1*, this study showed an important role of mitochondrial morphology and inner membrane cristae architecture in the effector functions of Th17 cells. However, *Opa1* deletion impaired proliferation in all effector T cell subtypes and increased cell death in Th1 and Treg cells, suggesting its broader impact on cell physiology. On the contrary, our study showed that TMEM11 deficiency did not influence development, differentiation, or proliferation of effector T cells [4]. Interestingly, even though both OPA1 and TMEM11 are widely expressed across all effector T cell subtypes, their loss impaired effector functions only in specific T cells, Th17 cells in the case of *Opa1* deletion and Th1 cells in the case of *Tmem11* deletion, suggesting cell-type specific functions of cristae components.

### Implications for T cell dysfunction in the tumor microenvironment

The link between cristae shape and mtROS is highly relevant to cancer immunology. TILs commonly exhibit mitochondrial swelling, cristae rarefaction, and elevated ROS, especially in nutrient-poor and hypoxic tumors [3,20]. Additionally, TILs exhibit impaired oxidative phosphorylation and mitochondrial depolarization, directly coupling cristae disruption to metabolic exhaustion. The metabolic and structural conditions imposed by tumors are strikingly analogous to those observed in TMEM11 deficiency. Tumors consume large quantities of glucose and amino acids, depriving T cells of substrates needed for optimal mitochondrial function [3]. Hypoxia forces T cells into inefficient metabolic states that amplify electron leak and mtROS generation. Chronic antigen stimulation elevates mitochondrial stress, altering dynamics, fusion–fission balance, and cristae organization [15,21]. Across these pressures, TILs often accumulate high levels of ROS and display fragmented or swollen mitochondria with poorly defined cristae. Recent studies of human TILs suggest that cristae disorganization can arise during tumor exposure and may precede or accompany transcriptional indicators of exhaustion, suggesting that mitochondrial architecture may be one of the first cellular structures to react to the tumor microenvironment [20,22,23]. Together, these observations suggest that cristae integrity represents a critical structural node in infiltrating T cells that both shapes and responds to metabolic and redox stress. Rather than positioning mitochondrial architectural disruption solely as either an upstream vulnerability or a downstream consequence, this framework supports a dynamic, feedback-driven model in which cristae remodeling can predispose T cells to dysfunction while also being exacerbated by ongoing metabolic stress. In this view, mitochondrial architecture operates upstream of, in parallel with, and potentially downstream from canonical inhibitory receptor pathways (PD-1, TIM-3, LAG-3), integrating structural and signaling cues that converge on T cell exhaustion [23].

Our findings suggest a possible molecular framework through which mitochondrial structural stress *could* be linked to functional decline. We hypothesize that elevated mtROS in TILs may promote diversion of acetyl-CoA away from histone acetylation, potentially dampening transcription of effector cytokines and chemokines required for effective tumor control. If operative, such epigenetic constraints could arise early during T cell infiltration and contribute to the progressive loss of function characteristic of exhaustion. Notably, because this putative mechanism would be rooted in mitochondrial architecture rather than canonical transcription factor signaling, it may help explain why exhausted T cells often exhibit limited responsiveness to signaling-based immunotherapies. In this context, we speculate that interventions such as checkpoint blockade or cytokine supplementation might be insufficient once mitochondrial architecture is compromised, as epigenetic responsiveness could be constrained by limited acetyl-CoA availability [17]. Tumor-associated metabolites, including lactate and long-chain fatty acids, may further exacerbate mitochondrial stress under hypoxic conditions, potentially destabilizing cristae structure and amplifying ROS accumulation [17,24,25]. Together, these considerations support a working model in which metabolic stressors converge to reinforce mitochondrial dysfunction, locking T cells into a high-ROS, low–acetyl-CoA state that may be difficult to reverse. Future studies will be required to directly test whether restoration of cristae integrity or acetyl-CoA homeostasis can reinvigorate exhausted TILs and improve immunotherapeutic efficacy. If validated, this model would expand current views of metabolic exhaustion to include mitochondrial architecture as a potentially reversible determinant of T cell dysfunction.

The possibility that cristae architecture influences T cell function raises broader, inherently speculative questions as to whether mitochondrial inner membrane organization could, in principle, represent a modifiable determinant of cellular fitness. In this conceptual framework, stabilization of cristae structure might preserve respiratory efficiency or limit mtROS-driven metabolic imbalance. However, proposed mechanisms — such as modulation of MICOS complex assembly or other factors governing cristae curvature [26] — are presented solely as illustrative examples rather than actionable therapeutic targets, as there is currently no direct translational evidence linking these proteins, including TMEM11, to cancer patient prognosis or responses to immune checkpoint blockade. Similarly, manipulation of acetyl-CoA routing as a means of restoring effector function remains conjectural in this context. Although acetate supplementation can enhance histone acetylation in metabolically stressed T cells [4], the relevance of this effect to mitochondrial architecture or exhausted TILs is still unknown. mtROS modulation represents another unresolved axis: while mitochondria-targeted approaches may offer greater specificity than generic antioxidants [27,28], whether such strategies can restore function without disrupting essential redox signaling remains an open question. Collectively, these considerations are intended to motivate future experimental investigation rather than suggest immediate therapeutic translation.

## Conclusion

In summary, our study identifies mitochondrial cristae architecture as a previously underappreciated determinant of effector T cell metabolism, epigenetic regulation, and cytokine output. By linking TMEM11-dependent maintenance of cristae integrity to mtROS homeostasis and acetyl-CoA allocation, we define a structural–metabolic axis that constrains Th1 effector function. These findings are particularly relevant in cancer immunology, where mitochondrial stress is pervasive, but they also advance a broader conceptual shift: mitochondrial architecture emerges not merely as a downstream consequence of metabolic stress, but as one of active regulators of immune function. Notably, TMEM11 loss perturbs redox balance and effector capacity without inducing overt mitochondrial failure, revealing how immune dysfunction can arise in the absence of compromised mitochondrial biogenesis or viability. Beyond cancer, this framework has implications for autoimmune disease, where excessive Th1 activity drives pathology. In such contexts, selective modulation of cristae integrity or acetyl-CoA flux toward lipid storage could, in principle, attenuate pathogenic cytokine production. Together, these observations position mitochondrial architecture as a context-dependent determinant of immune responsiveness across disease states.
